# Supplementary material for: Expertise-driven temporal gaze dynamics during anticipation in volleyball
Source: PLoS One. 2025 Oct 16;20(10):e0334702. doi: 10.1371/journal.pone.0334702 (PMC12530602; doi:10.1371/journal.pone.0334702)
Supplement: S6 Table — File including tables from complementary statistical analyses. (PDF) [file pone.0334702.s006.pdf]

Table A. Pairwise group comparison results for main fixation metrics.

	Phase:	Task:	Contrast:	*Estimate*	*SE*	*df*	*t-ratio*	*p*	*d*	*Sig.*
**Fixation Rate:**	Preparation	Prediction	Novice - Amateur	0.19	0.07	62.40	2.65	.031	0.24	*
			Novice - Expert	0.16	0.08	62.37	1.87	.099	0.19	
			Amateur - Expert	-0.04	0.08	62.37	-0.44	.660	-0.04	
		Control	Novice - Amateur	0.15	0.07	62.43	2.00	.149	0.18	
			Novice - Expert	0.09	0.09	62.51	1.10	.411	0.11	
			Amateur - Expert	-0.05	0.08	62.49	-0.66	.511	-0.06	
	Rally	Prediction	Novice - Amateur	0.10	0.07	62.25	1.33	.188	0.12	
			Novice - Expert	0.25	0.09	62.42	2.95	.013	0.31	*
			Amateur - Expert	0.15	0.08	62.44	1.90	.093	0.19	
		Control	Novice - Amateur	0.13	0.07	62.50	1.81	.113	0.16	
			Novice - Expert	0.18	0.09	62.53	2.10	.113	0.22	
			Amateur - Expert	0.05	0.08	62.50	0.57	.573	0.06	
**Fixation Duration:**	Preparation	Prediction	Novice - Amateur	-0.16	0.07	62.39	-2.41	.057	-0.26	
			Novice - Expert	-0.13	0.08	62.35	-1.74	.131	-0.22	
			Amateur - Expert	0.03	0.07	62.36	0.36	.719	0.04	
		Control	Novice - Amateur	-0.11	0.07	62.42	-1.60	.346	-0.17	
			Novice - Expert	-0.03	0.08	62.50	-0.38	.708	-0.05	
			Amateur - Expert	0.08	0.07	62.47	1.06	.440	0.13	
	Rally	Prediction	Novice - Amateur	-0.06	0.07	62.24	-0.89	.376	-0.10	
			Novice - Expert	-0.22	0.08	62.41	-2.90	.015	-0.36	*
			Amateur - Expert	-0.16	0.07	62.43	-2.25	.042	-0.27	*
		Control	Novice - Amateur	-0.12	0.07	62.49	-1.79	.118	-0.19	
			Novice - Expert	-0.16	0.08	62.52	-2.14	.108	-0.27	
			Amateur - Expert	-0.05	0.07	62.49	-0.63	.530	-0.07	

Significant Benjamini-Hochberg corrected comparisons are indicated with asterisks (*: $p < .05$).

Table B. ANOVA results for LMM analysis of saccade rate.

**Fixed Effect:**	***SumSq***	***MeanSq***	***df***	***F***	***p***	***Sig.***
Group	2.48	1.24	2, 55.02	4.30	.018	*
Task	0.07	0.07	1, 62.80	0.25	.617	
Phase	0.39	0.39	1, 60.92	1.36	.248	
Group × Task	3.96	1.98	2, 13884.98	6.87	.001	**
Group × Phase	10.66	5.33	2, 13884.22	18.50	< .001	***
Task × Phase	4.40	4.40	1, 57.74	15.28	< .001	***
Group × Task × Phase	2.21	1.11	2, 13884.91	3.84	.022	*

Type III ANOVA results with degrees of freedom calculations using Satterthwaite's method. Significant fixed effects are indicated with asterisks (*: $p < .05$; **: $p < .01$; ***: $p < .001$).

Table C. Pairwise group comparison results for saccade rate.

**Phase:**	**Task:**	**Contrast:**	***Estimate***	***SE***	***df***	***t-ratio***	***p***	***d***	***Sig.***
Preparation	Prediction	Novice - Amateur	0.24	0.07	63.98	3.54	.002	0.29	**
		Novice - Expert	0.20	0.08	63.97	2.64	.016	0.25	*
		Amateur - Expert	-0.03	0.07	64.01	-0.44	.662	-0.04	
	Control	Novice - Amateur	0.18	0.07	64.02	2.70	.027	0.22	*
		Novice - Expert	0.10	0.08	64.12	1.31	.287	0.12	
		Amateur - Expert	-0.08	0.07	64.12	-1.07	.287	-0.10	
Rally	Prediction	Novice - Amateur	0.10	0.07	63.82	1.50	.137	0.12	
		Novice - Expert	0.24	0.08	64.05	3.03	.010	0.28	*
		Amateur - Expert	0.13	0.07	64.10	1.83	.108	0.16	
	Control	Novice - Amateur	0.16	0.07	64.09	2.37	.032	0.19	*
		Novice - Expert	0.18	0.08	64.16	2.36	.032	0.22	*
		Amateur - Expert	0.02	0.07	64.13	0.32	.748	0.03	

Significant Benjamini-Hochberg corrected comparisons are indicated with asterisks (*: $p < .05$; **: $p < .01$).
