# Supplementary material for: Expertise-driven temporal gaze dynamics during anticipation in volleyball
Source: PLoS One. 2025 Oct 16;20(10):e0334702. doi: 10.1371/journal.pone.0334702 (PMC12530602; doi:10.1371/journal.pone.0334702)
Supplement: Attachment — Submitted filename: Response to Reviewers.docx [file pone.0334702.s008.docx]

Response to Reviewers

Concerning the revision of the manuscript *More efficient gaze behavior in relation to expertise during tactical decision-making in volleyball* (PONE-D-25-10199)

Dear Reviewers,

Dear Editors of PLOS ONE,

On behalf of my co-authors and myself, I would like to thank you for your thoughtful and constructive feedback on our manuscript. We hereby present to you our responses to all points that were raised and the subsequent changes we made to the manuscript. Please note that all mentions of specific line numbers (L. xx-xx) in our responses refer to the file “Revised Manuscript with Track Changes”.

With kind regards,

Thomas Kanatschnig

# Reviewer 1

*General comments:*

*The manuscript addresses a relevant and interesting topic related to perceptual-cognitive expertise in sport, focusing on gaze behavior in volleyball players of varying expertise levels. The use of a dynamic, domain-specific task and the inclusion of a temporally structured analysis (Preparation vs. Rally phases) are clear strengths of the study. The methodological approach, including eye-tracking and linear mixed effects modeling, is appropriate and generally well-described.*

*At the same time, several aspects of the study require clarification, expansion, or revision to strengthen its scientific contribution and improve methodological transparency. The following points outline the main areas where the manuscript could benefit from improvement.*

We would like to thank you for the detailed and constructive feedback on our manuscript. We deliberated on each point carefully and gave our best effort in tackling each of them thoroughly, as well as providing additional information to further strengthen our message. We hope that our revisions further support your positive impression of our work.

*1. Lack of an explicitly stated hypothesis*

*• While the introduction is coherent and based on relevant literature, it does not culminate in a clearly formulated research hypothesis or set of hypotheses. A testable hypothesis—derived from prior findings—would help frame the aims of the study and guide interpretation of the results, especially with regard to expected differences across groups, tasks, and phases.*

We agree and would like to thank you for making us aware of the fact that this aspect needs more information/clarification. First and foremost, we must clarify that the lack of specific testable hypotheses was a deliberate decision. A paragraph was added at the end of the Introduction section, which explains that because the analysis of gaze behavior was planned as a secondary explorative type of investigation for this study (stated as such in the preregistration for the research project, available at <https://osf.io/exhr3>), we have not defined specific a-priori hypotheses for the present analyses regarding gaze behavior. Instead, our approach was to examine expertise-related effects (novices vs. amateurs vs. experts) on gaze behavior in the framework of our experiment (tactical decision-making) and provide a comprehensive report of our findings for relevant gaze metrics (fixations, saccades). This is the reason why we backed away from formulating any concrete hypotheses for this work, however, for the sake of transparency we now made the explorative nature of this work clearer in order to avoid any misinterpretations regarding our research objectives (L. 103-106).

*2. Justification for the novelty of the study*

*• The topic of perceptual-cognitive expertise in volleyball is not new, and several previous studies have addressed gaze dynamics in this domain (e.g., Fortin-Guichard et al., 2020; Piras et al., 2014). The authors briefly mention some distinguishing aspects (e.g., inclusion of an intermediate group, temporal analysis), but a more explicit and focused explanation of what new insights this study provides would enhance the manuscript’s originality and relevance.*

Thank you for this suggestion. We added an explanation about what we deem to be the most important distinguishing factor of our investigation, which is the combination of four key methodological aspects of the experimental design, to provide a comprehensive picture of dynamic perceptual-cognitive processes underlying tactical decision-making in volleyball (L. 118-123).

*3. Methods section:*

*Unequal group sizes and sample power*

*• The three expertise groups are notably unbalanced (n = 14 experts, n = 25 amateurs, n = 19 novices). This raises concerns regarding the statistical power of the analysis, particularly for detecting interaction effects in the linear mixed models.*

We do see that the unequal group sizes can be considered as a limitation of our study. While we were able to reach our minimum targeted sample sizes for the Amateur and Novice groups (see next point regarding power calculation), the recruitment of professional volleyball players for the Expert group revealed to be difficult. Nevertheless, while we would have certainly preferred a more balanced sample, we are confident that our LMM results reflect reliable effects for two key reasons: (i) the high level of data quality we were able to achieve (L. 292-301), and (ii) the consistent patterns we observed across all gaze metrics, which would likely not have emerged had the LMM estimates been substantially biased due to sample imbalance. Moreover, LMMs are, in principle, designed to handle unbalanced data, making them arguably the most appropriate statistical approach for this scenario [1].

*• The manuscript does not report any a priori power analysis. The authors are encouraged to justify the sample size based on expected effect sizes or acknowledge this limitation explicitly in the discussion.*

Thank you for raising this important point. Regarding statistical power we must clarify, that due to the fact that our study in its core revolved around neurophysiological substrates of the relationship between sport expertise and efficiency (see [2]), our sample size calculations were based on studies investigating expertise-related effects of EEG frequency band power in the theta and alpha range [3]. Our a priori power calculations (see <https://osf.io/exhr3>) revealed that a minimum of 20-21 participants per group would be necessary to detect the expected effects in the respective EEG bands, which is what we were able to achieve in case of the Novice and Amateur groups (taking EEG data loss into account), but unfortunately fell short for the Expert group. While our power calculations were centered around the EEG analyses, we did find our sample/group sizes to be fairly comparable to those of previous investigations in the domain of perceptual-cognitive investigations relating to expertise. We are certainly far away from sample sizes of 27 [4] or even 62 [5] (here referring solely to the number of professional/expert individuals), yet the majority of the relevant literature that we refer to in our manuscript reports sample sizes of 10-15 individuals in a respective Expert group [6–9], which is why we think that our sample is comparable to these previous investigations with respect to statistical power. Furthermore, given our experimental design, we gathered multiple data points from each participant, which has been shown to improve statistical power [10]. Still, to acknowledge that a dedicated a priori power analysis for effects related to our investigated gaze metrics is lacking, we added a brief discussion of this point to the Limitations section (L. 546-552).

*Monocular eye tracking (right eye only)*

*• The use of monocular (right-eye) tracking is not uncommon in eye-tracking research. However, given the task’s emphasis on visual attention and the importance of peripheral vision in sport-specific scenarios, it would be helpful for the authors to justify this decision or at least discuss its implications.*

We agree that the use of binocular tracking could have provided more detailed information regarding depth perception and peripheral vision. The decision to use monocular tracking was made primarily for practical reasons, given its simpler calibration procedure, which aligned better with our co-registration design with EEG, as well as the deliberation that our primary gaze metrics of interest could be reliably captured using monocular tracking. Nevertheless, we acknowledge your comment and have now added this point to the Limitations section (L. 561-565).

*Statistical approach – appropriateness and clarity*

*• The use of linear mixed-effects models is appropriate for the study design. Nonetheless, the manuscript would benefit from:*

*• A clearer rationale for the choice of random effects structure,*

A more detailed explanation concerning the random effects structure of our models has now been added to the manuscript (L. 317-328).

*• A statement on whether model assumptions (e.g., normality, homoscedasticity) were checked and met,*

Model diagnostics have initially already been calculated to check for potential deviations from model assumptions and are reproducible alongside our main analyses by means of our provided analysis data and R code under “Supporting information”. Multicollinearity was generally found to be no concern, as generalized variance inflation factors [GVIF^(1/(2*Df))] of all fixed effects on each DV revealed values in an acceptable range [11]. Normality of residuals was checked on each level combination of Group, Task and Phase by inspection of skewness and kurtosis values, histograms, as well as Q-Q plots, which revealed deviations in a few instances, yet not to an extreme extent. Plotting residuals against fitted values to inspect potential issues with heteroscedasticity did not reveal any potential bias as scatter clouds generally followed a circular/oval pattern. Overall, we acknowledge that our data does not fit model assumptions perfectly in regard to normality, which is why we now included a sentence to the manuscript making aware of this circumstance (L. 335-337). However, the deviations we identified did not yield strong reasons for concern regarding the reliability of our estimations. Moreover, LMMs have been found to be quite robust to violations of assumptions, which according to Schielzeth et al. [1] “…should allow researchers to use mixed-effects models even if the distributional assumptions are objectively violated.”

*• More detail on how task block order was counterbalanced across participants.*

For the purposes of this response and the revisions made to the manuscript, we refer to the experimental design descriptions detailed in our previous publication Kanatschnig et al. [2]. Relevant sections from that publication will be quoted verbatim below. In the Materials section of our previous publication it is stated:

“…, the task was divided into two separate blocks. In the *Near-side* block the actions of interest (i.e., the setting in the Prediction condition and the service in the Control condition) occurred on the nearer side of the court (court side in front of the net), whereas in the *Far-side* block the actions occurred on the farther side of the court (court side behind the net), with respect to the camera’s field of view of the video recordings. The number of videos for the Prediction condition was 25 in the Near-side and 38 in the Far-side block. The videos of the Prediction condition in one block functioned as videos for the Control condition in the other block, given that the service always happened on the opposite court side. …”

Further, in the Design and general procedure section of our previous publication it is stated:

“… Before the first task block, participants carried out a practice run with different stimuli to get familiar with the task. Participants completed the first block, which was the Near-side block, in which the order of Prediction and Control conditions was counterbalanced across participants. After completing the Near-side block, participants carried out another training run for the second block, which was the Far-side block. During the Far-side block the order of Prediction and Control conditions was again counterbalanced. …”

In the first citation we describe the existence of a factor “Court” which describes on which side of the court (i.e., Near-side vs. Far-side) the respective action of interest depending on the task condition at hand (i.e., Prediction vs. Control) within each video occurred. This factor Court could however not be meaningfully investigated for reasons we discuss within the expanded description of our LMM random effect structure in the revised manuscript (L. 317-328), and it has therefore been omitted in the present investigation of gaze behavior. In the second citation we describe what the core of your comment refers to, namely the task block order and counterbalancing. It is stated here that there were two separate presentation blocks, Near-side and Far-side, referring to which court side the actions of interest occurred in the videos. All 63 videos occurred exactly one time per presentation block, only the task connected to the video changed depending on the respective block. Within each block the order of task conditions was counterbalanced across participants, i.e., one participant started the Near-side block with the Prediction condition, followed by the Control condition and then started the Far-side block again with Prediction, followed by Control, while another participant started each block with the Control condition followed by the Prediction condition. We now expanded the description of task block order and counterbalancing to provide a more detailed explanation regarding our procedure (L. 228-242); however, we did not include information regarding the omitted factor Court (Near-side vs. Far-side) from our previous publication [2], as we felt that it does not serve any additional information necessary to understand the basic principles of our present investigation and could therefore potentially distract the reader from more relevant information.

*4. Results: Missing analysis of prediction accuracy*

*• The main task required participants to anticipate the setter’s pass—a decision-making component that is central to the study’s rationale. However, the manuscript does not report participants’ accuracy in the Prediction condition. Including this data would provide essential behavioral context and allow stronger inferences about perceptual efficiency in relation to expertise.*

Thank you for this suggestion. We fully agree and have now added additional information regarding the behavioral results of the experiment, which (as stated in the manuscript) have been reported previously by Kanatschnig et al. [2] alongside the EEG analyses (L. 506-511).

*5. Limitations section is too brief*

*• The limitations section should be expanded to reflect important considerations that may affect the interpretation of results. In particular:*

*• The lack of analysis by player position, which is known to influence visual search behavior (Fortin-Guichard et al., 2020),*

*• The possibility of learning or repetition effects, as participants viewed the same stimuli in both task conditions,*

*• The use of monocular tracking and constrained head position, which may reduce ecological validity in a sport-performance context.*

Thank you again for these thoughtful observations. We have added justifications/discussions for each of them to the Limitations section (L. 539-580).

*Minor suggestions:*

*• Consider defining the concepts of “gaze anchors” or “visual pivots” more precisely and referencing foundational sources in this area.*

*• Lines 226–227 (Methods): the criteria for classifying saccades (velocity > 30°/s, etc.) should be supported with methodological references.*

These methodological aspects have now been adapted in the manuscript as well (L. 59-64; 276-278).

# Reviewer 2

*The authors present a study where they employed the temporal occlusion paradigm on video stimuli of national-level volleyball games (recorded from a bird's eye view, probably with a camera at the stand). The videos showed the scene from the preparation of a service "until the point before the setter plays the pass". In a (blocked, counter-balanced) “Prediction” condition, participants of three different expertise levels (19 novices, 25 amateur and 14 expert players) had to select one of four potential destinations of the setter’s pass. In a second “Control” condition, the task of the participants was simply to select one of three potential locations of the service player, a task without domain-specific knowledge requirements with respect to prediction of outcomes. Multiple gaze metrics (with respect to fixations and saccades) were recorded and analysed for the two time intervals before and after the service. In line with previous research, the authors found expertise-dependent differences for all groups (fewer fixations, longer fixation duration, fewer saccades), but only in the later time interval. In the first interval, only novices differed with comparatively higher rates of fixations and saccades, potentially indicating a more exporative gaze strategy of experts to gather situational knowledge and understanding already before the critical phase. The authors conclude that these findings emphasize the "importance of temporal dynamics" as well as a "comprehensive operationalization of perceptual-cognitive processes related to expertise".*

*While the authors seem follow a comprehensive and rigorous (statistical) analysis procedure, to me, the study design itself has several weaknesses, of which some seem not resolvable. Maybe this is due to the fact that the eye tracking part was only planned as "a secondary analysis [...] for the exploratory investigation of viewing behavior,[...] to look at the frequency of eye saccades and fixation times in specific regions of interest" (https://osf.io/exhr3). In the following, I will comment on four of theses weaknesses.*

We would like to thank you for your valuable as well as thorough review of our manuscript. We are pleased to see your favorable assessment of our analysis procedure and appreciate that you recognized key aspects we emphasized in our experimental setup, such as the inclusion of three groups with different levels of volleyball expertise, the incorporation of a control task, as well as the investigation of temporal dynamics in the form of different time intervals. In response, we aim to show that the methodological concerns you raised do not necessarily compromise the validity of our conclusions. We are confident that our findings represent a valuable contribution to the literature and hereby provide our detailed responses to your comments.

*First and foremost, the authors claim a "high ecological validity of the stimulus" (e.g., l. 469). However, the bird's eye view is obviously a very unnatural perspective for a player. While this perspective might be ok to assess coaches (for what the original material was intended as far as I understood), there is a substantial amount of evidence that perceptual expertise unfolds (best) in representative environments and task conditions. In the study at hand, the perspective on the visual information, the purely passive reception (even with constrained head position), but also the task (to predict the ball's final destination or to judge the position of the service player) and the way responses had to be given (by button presses) seem not to align with consequences arising from this evidence. If one assumes that a player actively engages with the environment (and, e.g., creates and manipulates perception by moving around, but also changes information intake/gaze behavior depending on the task at hand), my main concern is related to what the study results really tell us with respect to expertise differences in volleyball - and my critical answer would be "close to nothing". This becomes even more clear when considering that the gaze metrics analysed (apart from the descriptive gaze density plots) only relate to temporal aspects of gaze behavior independent from the location of the gaze and therefore are not related to the information in the scene but rather a generic aspect of oculomotor behavior when watching a volleyball serve. I am not sure how to restructure the manuscrupt to resolve this issue and to still extend to the currently available body of knowledge with respect to gaze behavior, but I would be open to further discuss ideas to overcome it with the authors.*

Thank you for your valuable observations. We believe that in response to this, it is important to clarify a few key points regarding the scope of our investigation:

(i) In response to your statement about the ecological validity of our stimulus material, we would like to indicate the importance of the context in which we referred to this aspect. We see that making the claim that stimulus material possesses high ecological validity without any further explanation can raise the idea that the stimulus presentation possesses ecological validity in every sense of the term, yet this is not what we claimed here. After stating that: “… we see our stimulus material to be of high ecological validity”, we then explicitly refer to the fact that our stimulus material: “… reproduces temporal dynamics of setting situations how they appear in real matches.”, i.e., our stimuli are dynamic real-world depictions (actual players acting in a competitive match) of actual tactical game situations (the depicted players have to make a genuine play decision) which the participant must judge. That being said, in this context, when investigating tactical game dynamics, we do see our claim of high ecological validity justified as we explicitly refer to the tactical component of our stimuli, which was our main focus of interest. Yet, in response to your concern we now rephrased this sentence in the revised manuscript to avoid any potential misinterpretations (L. 553-556).

(ii) Continuing with your argumentation regarding the general validity of our experimental design, we want to challenge your opinion that our design is not suited for the extraction of meaningful new information on expertise-related aspects of gaze behavior. In response to your argument: “While this perspective might be ok to assess coaches (for what the original material was intended as far as I understood), there is a substantial amount of evidence that perceptual expertise unfolds (best) in representative environments and task conditions”, we would like to emphasize that, while we agree that we think perceptual expertise has the highest potential to unfold in representative environments, this does not necessarily mean that specific aspects of perceptual expertise, such as tactical decision-making ability, are automatically best investigated in an naturalistic in-situ setting. Creating good task conditions that are representative in every respect is an enormous undertaking and the literature shows that compromises regularly have to be made in this regard. While, in-situ studies have the advantage of a higher situational-representative validity, their designs suffer from the fact that game sequences in a research setting do not have a real competitive character, as they usually follow predetermined scenarios. As described above, the biggest strength of our stimulus material is that it is derived from real competitive match conditions, which we consider to be a crucial advantage for the investigation of tactical decision-making (the core interest of our investigation). Moreover, it is common practice for professional athletes to perform video analysis, implying they are able to abstract tactical processes from an observer’s perspective in a video to an egocentric perspective for the purpose of decision-making. We believe that, as researchers, we must continue to rely on abstractions when investigating certain constructs of interest, and that the use of diverse methodological approaches should remain acceptable.

(iii) Finally, regarding your concern about a lack of novelty of our findings, we would argue that the incorporation of at least the three methodological aspects you mentioned in your general overview of our manuscript, namely a three-group design, inclusion of a control task, and the division into temporal intervals, are properties that are not commonly combined in one study (see L. 118-123). Moreover, with our design we were able to replicate, as well as extend upon previous findings related to perceptual-cognitive expertise in volleyball [6,7], by showing a general trend of higher expertise relating to higher efficiency on all investigated gaze metrics during the observation of dynamic rally sequences, as well as showing that this relationship “is not necessarily given during the entirety of the information extraction process” (see L. 513-515). While it is true that our study does not address an entirely novel research question, we believe it is important to emphasize the value of replicability, especially in light of the well-documented replication crisis affecting several empirical research domains [12,13]. In this context, we would also like to refer to PLOS ONE’s journal scope, which states that “PLOS ONE accepts scientifically rigorous research, regardless of novelty” (<https://everyone.plos.org/about-plos-one/>).

*Second, I have come across some potential issues with respect to the experimental design and analysis (some of which are only mentioned in the already published work ():*

Again, we would like to thank you for your thorough review and that you also addressed our previous publication [2]. You are correct in that there are certain methodological aspects that we did not present in its entirety in the present manuscript. The reason for that was to reduce the amount of information to the most important aspects that we felt were necessary to interpret the present results. We see that there exists the need for additional information regarding the points that you raised, which is what we want to provide in the following.

*- "At the end of the video, the last frame (immediately before the pass of the setter) was displayed to the participants for 0.5 s as a freeze frame, which was defined as the Freeze phase." --> This potentially affects gaze behavior as the temporal availability of the last frame's information is much longer compared to the real-life situation and therefore contrasts the idea of the temporal occlusion paradigm.*

The presentation of the last video frame for 0.5 s was a design decision for the investigation of neurophysiological substrates of tactical decision-making, as it is a commonly used method in EEG research (e.g., [14,15]) and therefore a consequence of our multimodal data acquisition approach. The so-called “Freeze” phase was not investigated in terms of gaze behavior as its short duration would not have provided enough fixation/saccade data to meaningfully analyze. This is why we decided not to describe the purpose of the Freeze phase in detail in the present manuscript, however we do inform the reader about the 0.5 s visibility of the last frame before the presentation of the response screen and have now added information regarding its purpose relating to the EEG analyses (L. 246-250). Regarding the effects of the presentation of still images/freeze frames, yes, we agree that this has potentially affected gaze behavior, as there is certainly the potential to extract additional information from this freeze frame in the 0.5 s of its presentation. However, we do not assume, that the existence of the freeze frame significantly impacted our results, considering the comparatively much longer and more information-rich preceding time intervals of interest (Preparation phase: ~5 s, Rally phase: 3s); moreover, a comparison between expertise groups should not be confounded by this aspect, as all participants were presented with the same experimental conditions. Nevertheless, to avoid any misinterpretations we now rephrased our wording in this regard (L. 170-173)

*- "Near side vs far side": in the original video set have been services from both teams - did you investigate them here as well? If so, I would expect differences in gaze behavior between these conditions due to the different visual angles which are covered.*

That is correct, and yes, we did investigate if there was an effect due to the factor Court (Near-side vs. Far-Side) in our data. Referring to the above given response to Reviewer 1’s comment about “how task block order was counterbalanced across participants”, the factor Court could not be meaningfully investigated as we discuss within the expanded description of our LMM random effect structure in the revised manuscript (L. 317-328): “For instance, we observed that fixation rates were systematically influenced depending on how near or far actions of interest within different levels of *Task* and *Phase* occurred across videos. The nearer/farther to the camera the observed actions happened, the higher/lower were generally fixation rates, presumably due to the generally wider/shorter visual angles of observed players, objects and distances between them, making it necessary to switch fixation positions more/less frequently. Therefore, by allowing the effect of *Task* and *Phase* as well as their interaction to vary across videos we specifically account for the occurrence of such random effects within our models.” As we stated in this excerpt, a direct comparison between the Near-side and Far-side conditions for our gaze metrics would have resulted in heavily biased results due to their underlying visual properties (e.g., due to different visual angles as you mentioned). Therefore, as we deemed the investigation of the factor Court to be uninformative in relation to gaze behavior, we decided not to provide any information on it in the present manuscript and put the overall focus on the comparisons between groups, task conditions and temporal intervals of interest.

*- What is exactly "the point before the setter plays the pass"? The last frame, before the setter touches the ball or when the ball leaves the hand of the setter? Please be more clear here.*

Thank you for raising this point, this sentence has now been adapted to include the exact information (L. 170-173).

*- Could the fixation cross in the screen's center affect the density plot or even the reported results? It seems they already fixate on a (potentially) information richt area and therefore could be biased in their natural gaze behavior. For better control, a fixation on a non information rich area at the edges of the screen seem advisable.*

The fixation point was centered in the middle of the screen primarily for calibration and quality check purposes. For that we oriented ourselves on the existing literature, as it can be considered a standard procedure in eye-tracking research (e.g., [5,16–18]). However, we are confident that such a bias towards the center of the screen at the beginning of stimulus presentation, did not significantly impact our results. We observed through visual inspection of individual gaze paths that participants tended to immediately fixate on specific players after stimulus onset, instead of leaving their gaze centered in the middle of the screen. Furthermore, we would argue that first observing the center of a game scene is actually what players would do in a real match as well, as for instance it would be quite unnatural for a player to fixate on a completely uninformative area during a real game.

*- How are the colors in the density plot scaled? How do differences in the amount of fixations across conditions affect the color / this plot?*

Thank you for this observation. You are correct that the color patterns are affected differently in the Preparation and Rally phase, as the general rate of fixations is affected by the length of a phase. We now added scaling legends to Figure 2 displaying the ranges of estimated probability densities for both phases, respectively. We also adapted the caption to include the additional information (L. 360-373). The base settings for the calculation of the density patterns, by means of the *stat_density_2d()* function are the same for both phases [stat_density_2d(aes(fill = after_stat(level)), geom = "polygon", alpha = 0.25, bins = 20)], the different density ranges are a result of the different total amounts of fixations performed in the respective phases.

*- No sample size calculations for eye tracking have been reported/performed. How did you come up with the sample sizes - can you also comment/discuss the varying amount of participants in the different groups?*

Thank you for this important observation as well. As the content of your question equals that of two above answered comments from Reviewer 1, please let us refer you to the responses to those comments above under: Reviewer 1 “3. Methods section: Unequal group sizes and sample power”

*- Why don't you report gaze behavior differences with respect to unsuccessful/successful trials, but rather only across groups?*

This is an interesting aspect and we thank you for raising it. Although this aspect of differences between successful and unsuccessful trials is an important consideration to make, we deliberately decided against taking this factor into account in our analyses for two main reasons. Firstly, we already knew that experts, amateurs and novices differed significantly from each other in behavioral task performance, i.e., the accuracies of their decisions in the Prediction condition of our task. These behavioral results have been reported (as stated in the manuscript) by Kanatschnig et al. [2]; from this publication we now included additional information to the present manuscript (L. 506-511): “On average experts achieved 67.0% task accuracy in Prediction, while amateurs and novices only achieved 45.1% and 35.8% accuracy, respectively. All three groups differed significantly from each other on task accuracy in Prediction, while no significant group differences were observed in Control (expert: 90.0%, amateurs: 90.3%, novices: 84.4%).” As the data show, there were significant group differences regarding the percentage of correctly answered trials, which is what we had hypothesized in our previous publication [2]. However, subdividing the groups gaze metric information into successful and unsuccessful trials would then have resulted in considerable imbalance in our data between those two conditions across groups. While we acknowledged that LMMs are quite robust against data imbalances [1], in this case we fear that it would impose considerable systematic bias on our estimations. Which brings us to the second reason, which is that we wouldn’t have wanted to further increase the complexity of our LMM structure, thereby increasing the risk of overfitting. Nevertheless, in response to your comment, we went back and looked at the data and compared the distributional patterns (i.e., boxplots) and expanded the plots by subdividing between the response categories “1” (= successful trial) and “0” (= unsuccessful trial) and found that overall, there were no major differences observable between those two categories across the different factor levels of our analysis. You may investigate these plots via the attached file “REVIEW_ONLY.zip” (Note: these plots do not include brackets marking statistically significant differences, as LMMs were not calculated for the reasons described above).

*Third, there are several mentions of expertise-dependent efficiency differences when extracting information during fixations. To me, these arguments seem purely speculative without taking the relative locations of the fixation into account. So also this line of argumentation needs substantial rework and better justification.*

*And fourth and finally, also aspects with respect to the use of peripheral information can only properly discussed when analysis which information is visible in which peripheral eccentricity and whether it can be used to inform prediction and/or decision making.*

It is correct that our findings do not give detailed insight about exact fixation locations, i.e., areas of interest (AOI). This is a shortcoming we now added to the Limitations section of the manuscript, as we agree that it is an important aspect (L. 565-567). We also agree that our discussion regarding the use of peripheral vision must be seen as an interpretation rather than factual evidence. However, the literature shows that dedicated AOI analyses usually leave room for interpretation as well, e.g., [6,7]. We consider our interpretations – grounded in findings from previous research – to be valid reflections on the causes of the expertise-related effects we observed. Nonetheless, we have revised our wording to clarify our theoretical approach (L. 526-538).

*I think at the current stage, more detailed feedback on the manuscript is not needed - to me these four issues need to be resolved first and this will probably cause a major rework of the manuscript's structure.*

To conclude, we again would like to express our sincere appreciation for the valuable feedback you provided on our manuscript. We are thankful for your thorough observations, which lead us to improve and rework our manuscript, albeit not to such a major extent as you have pointed to. We already ensured before submission that we handled our data acquisition and analysis procedures to the highest possible standards. We now consider also our manuscript to reflect these high standards. As initially mentioned, we hope that through our responses we were able to convey that we deem our findings to be a valuable addition to the current literature.

# Journal Requirements

*1. Please ensure that your manuscript meets* *PLOS ONE's style requirements, including those for file naming. The PLOS ONE style templates can be found at*

*https://journals.plos.org/plosone/s/file?id=wjVg/PLOSOne_formatting_sample_main_body.pdf and*

*https://journals.plos.org/plosone/s/file?id=ba62/PLOSOne_formatting_sample_title_authors_affiliations.pdf*

PLOS ONE's style requirements were adopted.

*2. We note that the grant information you provided in the ‘Funding Information’ and ‘Financial Disclosure’ sections do not match.*

*When you resubmit, please ensure that you provide the correct grant numbers for the awards you received for your study in the ‘Funding Information’ section.*

This work was financed in its entirety by the University of Graz. Therefore, there is no external funding information to be provided for this work, as no grant or similar financial support has been received for it.

*3. Thank you for stating the following in the Acknowledgments Section of your manuscript:*

*“This study was supported by the Field of Excellence COLIBRI (Complexity of Life in Basic Research and Innovation, University of Graz).”*

*We note that you have provided funding information that is currently declared in your Funding Statement. However, funding information should not appear in the Acknowledgments section or other areas of your manuscript. We will only publish funding information present in the Funding Statement section of the online submission form.*

*Please remove any funding-related text from the manuscript and let us know how you would like to update your Funding Statement. Currently, your Funding Statement reads as follows:*

*“This study was financially supported by the University of Graz for coverage of publication fees. No additional external funding was received for this study.”*

*Please include your amended statements within your cover letter; we will change the online submission form on your behalf*

As mentioned above, all financial support received for this work was provided by the University of Graz. The Field of Excellence “COLIBRI” is a doctoral consortium aimed at the amplification of interdisciplinary scientific collaboration within the University of Graz, which the authors TK, MT and SK are members of. COLIBRI was not directly involved financially in this work but served indirectly as a networking platform for the authors, for which they wanted the consortium’s involvement to be acknowledged. Therefore, we assume that the current Funding and Acknowledgments statements we provided appropriately serve their intended purposes. If that is not the case, we would kindly ask the editorial office to please follow up with us in this regard.

*4. We note that Figures 1 and 2 in your submission contain copyrighted images. All PLOS content is published under the Creative Commons Attribution License (CC BY 4.0), which means that the manuscript, images, and Supporting Information files will be freely available online, and any third party is permitted to access, download, copy, distribute, and use these materials in any way, even commercially, with proper attribution. For more information, see our copyright guidelines: http://journals.plos.org/plosone/s/licenses-and-copyright.*

*We require you to either (1) present written permission from the copyright holder to publish these figures specifically under the CC BY 4.0 license, or (2) remove the figures from your submission:*

*a. You may seek permission from the original copyright holder of Figures 1 and 2 to publish the content specifically under the CC BY 4.0 license.*

*We recommend that you contact the original copyright holder with the Content Permission Form (http://journals.plos.org/plosone/s/file?id=7c09/content-permission-form.pdf) and the following text:*

*“I request permission for the open-access journal PLOS ONE to publish XXX under the Creative Commons Attribution License (CCAL) CC BY 4.0 (http://creativecommons.org/licenses/by/4.0/). Please be aware that this license allows unrestricted use and distribution, even commercially, by third parties. Please reply and provide explicit written permission to publish XXX under a CC BY license and complete the attached form.”*

*Please upload the completed Content Permission Form or other proof of granted permissions as an "Other" file with your submission.*

*In the figure caption of the copyrighted figure, please include the following text: “Reprinted from [ref] under a CC BY license, with permission from [name of publisher], original copyright [original copyright year].”*

*b. If you are unable to obtain permission from the original copyright holder to publish these figures under the CC BY 4.0 license or if the copyright holder’s requirements are incompatible with the CC BY 4.0 license, please either i) remove the figure or ii) supply a replacement figure that complies with the CC BY 4.0 license. Please check copyright information on all replacement figures and update the figure caption with source information. If applicable, please specify in the figure caption text when a figure is similar but not identical to the original image and is therefore for illustrative purposes only.*

The captions of Figures 1 and 2 have been modified to include the appropriate licensing statement for the reprinted material. As the author TK is the original owner and creator of the copyrighted material, we were informed by the PLOS ONE journal office that a permission for reuse is not necessary in this case.

*5. We note that there is identifying data in the Supporting Information file <**S1_Data.csv>. Due to the inclusion of these potentially identifying data, we have removed this file from your file inventory. Prior to sharing human research participant data, authors should consult with an ethics committee to ensure data are shared in accordance with participant consent and all applicable local laws.*

*Data sharing should never compromise participant privacy. It is therefore not appropriate to publicly share personally identifiable data on human research participants. The following are examples of data that should not be shared:*

*-Name, initials, physical address*

*-Ages more specific than whole numbers*

*-Internet protocol (IP) address*

*-Specific dates (birth dates, death dates, examination dates, etc.)*

*-Contact information such as phone number or email address*

*-Location data*

*-ID numbers that seem specific (long numbers, include initials, titled “Hospital ID”) rather than random (small numbers in numerical order)*

*Data that are not directly identifying may also be inappropriate to share, as in combination they can become identifying. For example, data collected from a small group of participants, vulnerable populations, or private groups should not be shared if they involve indirect identifiers (such as sex, ethnicity, location, etc.) that may risk the identification of study participants.*

*Additional guidance on preparing raw data for publication can be found in our Data Policy (https://journals.plos.org/plosone/s/data-availability#loc-human-research-participant-data-and-other-sensitive-data) and in the following article: http://www.bmj.com/content/340/bmj.c181.long.*

*Please remove or anonymize all personal information, ensure that the data shared are in accordance with participant consent, and re-upload a fully anonymized data set. Please note that spreadsheet columns with personal information must be removed and not hidden as all hidden columns will appear in the published file.*

Thank you for bringing this to our attention. In the updated version of the “S1_Data.csv” file, we have replaced the content of the “age” and “sex” columns with NAs for all participants to prevent the risk of re-identification. We refer to this modification in the “S5_Code.R” file in the respective analysis sections for age and sex, stating that this data has been “blurred for privacy reasons”, while still providing the analysis code from which the results within Table 1 of the manuscript originate. We hope that through these adjustments our data complies with PLOS ONE's Data Policy. If that is not the case, we would kindly ask the editorial office to please follow up with us in this regard.

# Additional Changes

- Minor general changes concerning grammar and formatting were performed to the main text.
- The position of Figures/Tables as well as text that reference them have been adapted to comply with formatting guidelines.
- The formatting of Figure 1 as well as its caption text have been reworked
- The formatting of Figure 2 as well as its caption text have been reworked. No changes were made regarding the depicted density patterns.
- The file “S5_Code.R” now also includes the visualization code for the replication of statistical plots (boxplots, heatmaps) from Figures 2-5.

# References

1.	Schielzeth H, Dingemanse NJ, Nakagawa S, Westneat DF, Allegue H, Teplitsky C, et al. Robustness of linear mixed‐effects models to violations of distributional assumptions. Sutherland C, editor. Methods Ecol Evol. 2020;11: 1141–1152. doi:10.1111/2041-210X.13434

2.	Kanatschnig T, Schrapf N, Leitner L, Wood G, Körner C, Tilp M, et al. EEG theta and alpha oscillations during tactical decision-making: An examination of the neural efficiency hypothesis in volleyball. PLoS One. 2025;20: e0318234. doi:10.1371/journal.pone.0318234

3.	Filho E, Dobersek U, Husselman T-A. The role of neural efficiency, transient hypofrontality and neural proficiency in optimal performance in self-paced sports: a meta-analytic review. Exp Brain Res. 2021;239: 1381–1393. doi:10.1007/s00221-021-06078-9

4.	Afonso J, Garganta J, McRobert A, Williams AM, Mesquita I. The perceptual cognitive processes underpinning skilled performance in volleyball: Evidence from eye-movements and verbal reports of thinking involving an in situ representative task. J Sport Sci Med. 2012;11: 339–345.

5.	Fortin-Guichard D, Laflamme V, Julien A-S, Trottier C, Grondin S. Decision-making and dynamics of eye movements in volleyball experts. Sci Rep. 2020;10: 17288. doi:10.1038/s41598-020-74487-x

6.	Piras A, Lobietti R, Squatrito S. A study of saccadic eye movement dynamics in volleyball: Comparison between athletes and non-athletes. J Sports Med Phys Fitness. 2010;50: 99–108.

7.	Piras A, Lobietti R, Squatrito S. Response Time, Visual Search Strategy, and Anticipatory Skills in Volleyball Players. J Ophthalmol. 2014;2014: 1–10. doi:10.1155/2014/189268

8.	Piras A, Raffi M, Perazzolo M, Malagoli Lanzoni I, Squatrito S. Microsaccades and interest areas during free-viewing sport task. J Sports Sci. 2019;37: 980–987. doi:10.1080/02640414.2017.1380893

9.	Vansteenkiste P, Vaeyens R, Zeuwts L, Philippaerts R, Lenoir M. CUE USAGE IN VOLLEYBALL: A TIME COURSE COMPARISON OF ELITE, INTERMEDIATE AND NOVICE FEMALE PLAYERS. Biol Sport. 2014;31: 295–302. doi:10.5604/20831862.1127288

10.	Baker DH, Vilidaite G, Lygo FA, Smith AK, Flack TR, Gouws AD, et al. Power contours: Optimising sample size and precision in experimental psychology and human neuroscience. Psychol Methods. 2021;26: 295–314. doi:10.1037/met0000337

11.	Fox J, Monette G. Generalized Collinearity Diagnostics. J Am Stat Assoc. 1992;87: 178. doi:10.2307/2290467

12.	Aarts AA, Anderson JE, Anderson CJ, Attridge PR, Attwood A, Axt J, et al. Estimating the reproducibility of psychological science. Science (80- ). 2015;349. doi:10.1126/science.aac4716

13.	Korbmacher M, Azevedo F, Pennington CR, Hartmann H, Pownall M, Schmidt K, et al. The replication crisis has led to positive structural, procedural, and community changes. Commun Psychol. 2023;1: 3. doi:10.1038/s44271-023-00003-2

14.	DeCouto BS, Smeeton NJ, Williams AM. Skilled Performers Show Right Parietal Lateralization during Anticipation of Volleyball Attacks. Brain Sci. 2023;13: 1204. doi:10.3390/brainsci13081204

15.	Denis D, Rowe R, Williams AM, Milne E. The role of cortical sensorimotor oscillations in action anticipation. Neuroimage. 2017;146: 1102–1114. doi:10.1016/j.neuroimage.2016.10.022

16.	Foulsham T, Kingstone A. Fixation-dependent memory for natural scenes: An experimental test of scanpath theory. J Exp Psychol Gen. 2013;142: 41–56. doi:10.1037/a0028227

17.	Rayner K, Smith TJ, Malcolm GL, Henderson JM. Eye Movements and Visual Encoding During Scene Perception. Psychol Sci. 2009;20: 6–10. doi:10.1111/j.1467-9280.2008.02243.x

18.	Walcher S, Korda Ž, Körner C, Benedek M. The effects of type and workload of internal tasks on voluntary saccades in a target-distractor saccade task. Wang J, editor. PLoS One. 2023;18: e0290322. doi:10.1371/journal.pone.0290322
