# Supplementary material for: Expertise-driven temporal gaze dynamics during anticipation in volleyball
Source: PLoS One. 2025 Oct 16;20(10):e0334702. doi: 10.1371/journal.pone.0334702 (PMC12530602; doi:10.1371/journal.pone.0334702)
Supplement: Attachment — Submitted filename: Response_to_Reviewers_auresp_2.docx [file pone.0334702.s009.docx]

Response to Reviewers

Concerning the revision of the manuscript: *More efficient gaze behavior in relation to expertise during tactical decision-making in volleyball* (PONE-D-25-10199R1)

Dear Reviewers,

Dear Editors of PLOS ONE,

On behalf of my co-authors and myself, I would like to thank you again for your valuable feedback on our manuscript. We hereby present to you our responses to all points that were raised and the subsequent changes we performed on the manuscript in the second round of revision. Please note that all mentions of specific line numbers (L. xx-xx) in our responses refer to “Revised Manuscript with Track Changes” (DOCX/PDF).

With kind regards,

Thomas Kanatschnig

# Reviewer 1

*The authors addressed most of the comments and implemented the changes that were feasible. I appreciate the effort they put into the revision. I have no further comments.*

We thank Reviewer 1 for their positive evaluation and insightful feedback on our manuscript.

# Reviewer 2

*I pointed out 4 key issues in my previous review, which did render more detailed feedback on the manuscript superfluous at that stage. While the authors tried to address these in their revised work and the rebuttal, I am still not convinced that the submitted work provides valuable insights. I will try to be more concise this time and add details with respect to the content rather than the methodological aspects only.*

We sincerely thank you, Reviewer 2, for your thoughtful and detailed review of our manuscript. We greatly appreciate the time you took to assess our work. In the following, we aim to address the additional points you raised, clarifying the scope and intention of our work, and outlining why we believe it contributes valuable insights to the literature.

*l. 39f. You call your task a “tactical decision-making task”. I would rather argue that the task is an “observation and prediction task”. This notion is important as tactical decision making implies that the participant is engaged in the situation and tries to solve it, while in your setting the person is only observing movement patterns (related to my previous review: in unnatural viewing conditions for players) and trying to predict the setter’s target player.*

Thank you for this observation. We agree that, since participants were not able to make an active tactical decision in the experimental situation, the term prediction provides a more accurate description. We have revised the task description throughout the manuscript, replacing “tactical decision-making” with “volleyball anticipation”. We kept the two task condition labels “Prediction” and “Control”, as the term prediction is already in use there for describing our main task condition.

*l.59f. Gaze anchors and visual pivots are only meaningful if analysed with respect to the underlying SPATIOtemporal aspects of the stimuli. A purely temporal analysis cannot unveil these aspects (it could be, that more experienced persons just “know” where to look at and saccade directly to that stimulus (as you pointed out in l.68f), which would directly result in lower amounts of saccades and potentially longer fixations). This already calls for an AOI-based analysis of your data – which you already pointed out in your preregistration (“For the exploratory investigation of viewing behavior, we plan to look at the frequency of eye saccades and fixation times in specific regions of interest.”) and inferred its necessity in a later section of your introduction (l.88f). The narrowing down to fixation and saccade rates (l.95f.) is not substantiated at all.*

We appreciate your thoughtful evaluation and fully agree that incorporating spatial aspects of gaze behavior could provide an even clearer picture of the perceptual-cognitive processes during setting prediction. As you noted, our preregistration initially included plans for an area-of-interest (AOI) analysis. However, during data inspection we realized that the camera angle – chosen to capture the entire playing field and players to provide as much information possible for setting prediction – did not allow for reliable AOI coding. In many sequences, players were partially or fully occluded by other players, especially during the Rally phase. Gaze fixations could therefore not be uniquely attributed to specific players or interest areas. Given that, we decided that a spatial analysis would risk introducing misleading conclusions. Instead, we focused specifically on the temporal aspect of gaze behavior, where our design allowed us to contribute new insights, e.g., by comparing three expertise levels, including a control condition, and using real-life match recordings as stimuli. In doing so we both replicated prior findings and extended them with previously underexplored aspects of temporal gaze dynamics. We have now expanded this part in the Limitations section (L. 599-604) to explicitly address the absence of a spatial analysis and to clarify why we concluded that it was not feasible in the present study. We hope this demonstrates our awareness of the issue and our intention to inform future work in this direction.

*l.64f. How does Faubert’s 3D-MOT task fit into the line of argumentation here?*

Our reference to the 3D-MOT task was meant to signify the superiority of professional athletes in adapting to learn and perform during dynamic visual scene observation tasks. Certain core features of the 3D-MOT, such as the necessity of: “… distributing attention among a number of moving targets among distractors… “ [1] are very similar to our task, in which it is likewise necessary to distribute the attention among multiple targets, i.e., the players in our scenario. Therefore, we saw the connection fitting, albeit we see that we did not communicate this point well enough. We have now extended this segment in the manuscript (L. 68-71).

*l.101f. This is the first mention of the control task. To me, this control task is just a completely different task with clearly differing visual demands (e.g. focusing on one player only vs. a “tactical situation”). The rationale for incorporating this task does not become clear. To me, a task which purely manipulates domain knowledge requirements while keeping perceptual requirements identical would be the only fair comparison. Moreover, if employing standardized lab-based experiments, you should respect the max-con-min principle; it is not clear at all why you alter the amount of response options between experimental and control task (3 vs 4, see also l. 193f.). Is it correct that the videos also differed between the experimental and control task for the same condition as you defined the “action of interest” to be on the near or far side of the net (l. 231f.)? If so, this additionally confounds your experimental manipulation.*

We gladly want to clarify the rationale for the Control condition. In the revised manuscript we now provide a more detailed description of this condition (L. 105–109). Following the definition you provided, it was also our intention to design a control task that manipulates domain knowledge requirements while keeping perceptual demands identical. To this end, we presented the same videos in both conditions, varying only the action of interest: participants predicted the setting outcome in the Prediction condition (domain knowledge beneficial) and identified the service location in the Control condition (domain knowledge not required). We acknowledge that the difference in response options (3 vs. 4) introduces some variance between the two conditions; however, because gaze behavior was measured only during scene observation, i.e., Preparation and Rally phase, and not during the Response phase, we consider this unlikely to have affected our main gaze-related dependent variables. Regarding the number of videos showing the respective actions of interest on each court side (i.e., Near-side and Far-side), in the Prediction condition there were 25 videos in which the setting happened on the Near-side and 38 videos in which it happened on the Far-side of the court, meaning that there is an uneven number of instances where the respective actions of interest (i.e., setting and service) happened on the respective court sides. While ideally the number of videos would have been balanced, this affected all participants equally. Also, to test whether the numbers of videos per condition were significantly different from a uniform distribution, a chi-square test was calculated which yielded a non-significant result (*χ*^2^(1) = 2.68, *p* = .102), implying that a potential confounding effect of the variance in video counts might only be marginal.

*l.103f. This section is important but for me way too generic to be meaningful. A good introduction should set the stage, follow a funnel-like structure (narrowing down) and result in a clear-cut research question (even if you performed an exploratory design).*

*l. 107f. Parts of this section seem to belong to the introduction.*

We have now revised these sections (L. 100-138).

*l. 166f. You showed women’s indoor volleyball situations to males and females but you did not mention this potentially confounding aspect. It is expected that domain-specific expertise unfolds (best) when confronted with situations the participants have experienced a lot – which is not true for men observing women’s games. This argument again can be extended with respect to the bird’s eye perspective (and even from the “wrong side” when confronted with the “FAR” condition) – but I raised this point during the first phase of the review already. At least a discussion with respect to higher (and unnatural) task demands (e.g., as you need to mentally rotate) compared to the real situation and their effects on your measures seems necessary.*

Thank you for reiterating these important points. We have now incorporated this into the Limitations section (L. 604-610).

*l. 177f. I do not find a discussion of the varying duration of the preparation phase – from 3 to 10 seconds is quite a range which might influence gaze behavior (e.g., triggering exploratory viewing behavior purely because of longer durations).*

A discussion of this aspect has now been added to the manuscript (L. 195-197).

*l. 179f. It is not clear how a natural game situation can be “fixed” to exactly 3 seconds. There must be some time variation from service to the ball contact of the setter.*

We recognize that this point required adjustment. It is correct that there is a certain degree of variation in the lengths of the rallies. We have now rephrased this section to make this circumstance clearer (L. 200-202). Our videos show matches between women's teams from the same league where rallies are very homogeneous, i.e., very similar in terms of playing speed, ball height, and ball trajectory/distance. The 3 second time interval, therefore, very accurately covered the rally in each respective video and no substantial distortion of the results due to the fixed time interval was expected.

*l. 247f. You argued in your rebuttal that the 500ms static image will not influence the viewing behaviour during the preparation and rally phase. I strongly disagree here as experts are very efficient in extracting relevant information (as you pointed out in your introduction). So, within a couple of trials I expect that experts only focus on extracting the information in the last 500ms – maybe with an exploratory viewing behaviour to optimally locate the gaze to the most information rich area. This would mean that the observed gaze behaviour difference during rally is completely irrelevant for them to solve the task. This is a fundamental flaw of the experiment, and you can only get (exploratory) insights when doing spatiotemporal analyses. This aspect has also consequences for your discussion (l. 454f) rendering these insights potentially meaningless for solving the task.*

Thank you for raising this important concern. We agree that the Freeze phase could, in principle, provide relevant information, which is why we initially examined it. However, given its short duration (500 ms), almost no unique fixations occurred in this phase, which is why we did not report it in the manuscript. To demonstrate this, we extended our main analysis file “S5_Code”, which now includes additional analysis of the Freeze phase. The Freeze phase analysis of fixation count (not rate, as this phase was only 500ms) shows a similar picture to that of the Rally phase. However, the interpretability of these results is impaired, as mentioned above, due to the short duration of the phase, which resulted in low fixation counts. Furthermore, we saw a floor effect since multiple participants recorded on average only one fixation in this phase, which negatively affected model integrity. To directly address your point regarding the relevance of the Preparation and Rally phases, we now additionally compared gaze behavior between phases within each group. If experts relied exclusively on the Freeze phase, one would expect largely random viewing patterns in the preceding Preparation and Rally phases, with no systematic differences across them. Our newly added pairwise comparisons (added to the main analysis file “S5_Code”), however, show that experts exhibited the strongest differences between Preparation and Rally on our main dependent measures (i.e., fixation rate, fixation duration) in the Prediction condition, with the largest effect sizes of any group. This suggests that their gaze behavior during these phases is not irrelevant but rather reflects an adaptive allocation of attention over the course of video observation. To inspect the results of these additional analyses, please see the file “REVIEW_ONLY_2”. The results can also be reproduced using the available data and analysis code under Supporting information.

*l.266f. There was a large initiative in establishing a reporting guideline for eyetracking experiments (Dunn et al. 2023, https://doi.org/10.3758/s13428-023-02187-1), which tries to make eye tracking experiments more replicable and understandable for other researchers. This guideline should be followed. Moreover, the reference (Walcher et al. [36]) seems not to employ a standard way of classifying gaze into fixations and saccades – which contributes to confusion with respect to terminology (a fixation is something different than a smooth pursuit eye movement).*

This is very valuable insight. We have revised the Methods section to align with the reporting guidelines of Dunn et al. [2], ensuring that all relevant aspects are now addressed (L. 139–141; 237-320). For the classification of fixations and saccades, we followed a procedure that has been shown to yield reliable results in previous studies [3–5]. Regarding potential confusion about the differentiation between fixations and smooth pursuit movements, we have rephrased the relevant segment for greater clarity (L. 294-298).

*l. 321f. Your rationale for constructing the model emphasizes the point I already made in my prior review that viewing conditions are critical and systematically affect the gaze behavior observed. In my view, this is very critical. Turning it to the positive side: Maybe one could use this (unintended) manipulation of stimulus demands (having information in smaller or larger ranges of visual angles) to explore gaze behavior differences between near and far situations. The current way of analysis, however, weakens the findings.*

This is an important consideration, and we thank you for raising it. We fully agree that viewing conditions and stimulus demands affect gaze behavior. We constructed a linear mixed-effect model to explicitly capture variance arising from differing visual features of video stimuli, as well as from changing viewing conditions following phase transitions. In the Statistical Analysis section, we clarify relevant considerations regarding statistical implications (L. 335-352).

*l.337f. Can you be clearer about the effect of employing Benjamini-Hochberg adjustments to your stats?*

Thank you for this suggestion. We expanded the description of the Benjamini-Hochberg adjustment in the Statistical analysis section, clarifying the effect of the Type I error correction method on our pairwise comparisons (L. 361-363).

*l.360f. Figure heading: the coloring of the graph still seems misleading as one cannot compare between conditions (i.e. the same lightness means different things due to varying amounts of data). I think you should standardize this to help the reader understand and compare the graphs. In addition, these graphs only take on condition and neglect the other – so I guess it would be informative to see gaze densities but maybe also scan-paths for all conditions (to get a full picture of the viewing pattern).*

Thank you for this remark regarding Figure 2. We would like to clarify that the projected gaze distribution patterns are comparable across the Prediction and Control conditions, as they are standardized within each phase. As the fixation counts were similar between conditions within a phase, this approach was found to be most appropriate for comparison. However, fixation counts varied across phases due to their different durations. Standardizing across phases would therefore have created a bias, as the higher number of fixations in one phase would overshadow those in the other phase and distort the distribution. We recognize that our figure caption might have been misleading in that regard, so we adapted it to provide more clarity (L. 392-394).

*l. 387f./401f. Please refrain from using “fixation rate” and “fixation duration” for the time intervals in which the participants can extract information from the visual scene – this is something different!*

We made sure to keep the revised manuscript free from such instances.

*To sum up, due to the points raised in my previous review and now here in more detail I do not see the authors’ interpretations and conclusions justified. Maybe the data set might still provide interesting hints for future research when analysed in a clever (e.g., turning experimental design flaws into interesting aspects) and spatiotemporal (including AOI-based, but also more fine grained temporal analyses (e.g., separating the last 500ms from the rest of the rally)) way.*

We thank you again for your careful evaluation and for sharing your perspective. We understand your concerns regarding the interpretation of our findings and acknowledge the limitations you highlighted. At the same time, we believe that our temporal analyses, despite these constraints, provide meaningful insights into expertise-related gaze behavior and extend prior work in this field. Following your suggestions, we have emphasized the methodological limitations more clearly in the manuscript and highlighted directions for future research.

# Reviewer 3

*The paper reports on an eye tracking experiment conducted with novice, amateur and expert volleyball players. Although not presenting entirely novel findings, this was acknowledged by the author, and the paper still adds some insights to the known literature, as well as confirming some previous findings.*

*The authors already replied to the comments of two reviewers, which improved the manuscript. I would like to see this article published, but I think there needs to be some further clarification first. Below some additional comments to those already made by the other reviewers.*

Thank you, Reviewer 3, for your valuable feedback. Requested clarifications and subsequent modifications to our manuscript are discussed in the following.

*Comment #1*

*The title does not suit the content of the paper. The task in the experiment is to predict the direction of the pass. This is not tactical decision-making, it is pattern recognition and (mostly) anticipation. The participants do not need to make a decision (e.g. what is the best course of action), they need to predict. So I believe it is not suitable to refer to this experiment as decision making nor as tactical.*

Thank you, as this was also pointed out by Reviewer 2, we see it as a valid argumentation and acknowledge that in general our task can be better described as an anticipation/prediction task. We therefore changed the manuscript title to “Expertise-driven temporal gaze dynamics during anticipation in volleyball”. Also, following your interpretation and that of Reviewer 2, we removed the “tactical” aspect from our task description and rephrased this aspect accordingly throughout the manuscript.

*Comment #2:*

*Please refrain from using the wording ‘fewer fixations of longer duration’, as this could mean both that experts made fewer long fixations, or that they made fewer fixations but they were of longer duration. This also occurs a couple of times in the discussion.*

*See: Vansteenkiste, P. (2022). Fewer fixations of longer duration can lead to more fixations of longer duration: A commentary on the description of the visual behaviour of expert performers in sports. German Journal of Exercise and Sport Research, 52(1), 198-199.*

This is a crucial aspect that we have overlooked so far, thank you. Text passages with this type of wording have been rephrased accordingly.

*Comment #3:*

*It is not clear to me why both Fixation rate and Saccade frequency is included in this study. Shouldn’t fixation rate and saccade rate be the same, assuming that a fixation is always followed by a saccade? In what case could fixation rate and saccade frequency be different? Unless I’m mistaking, I believe this metric (saccade frequency) could (and should) be omitted from the paper.*

Thank you for this suggestion. Generally speaking, we agree that fixation rate and saccade rate are innately connected and can even be described as the same. From a psychometric standpoint, we would argue that it makes a difference which one of the two is being inspected, depending on how they are defined/counted (e.g., during phase transitions). Nevertheless, we agree that the analysis of saccade rate in this case does not add highly unique information, which is not already provided through the analysis of fixation rate. Yet, as the definition of saccades has an integral connection to the definition of fixations, and because the investigation of saccades was a core part of our analysis, we felt it would be necessary not to omit this part entirely. Therefore, we decided to move all saccade-related statistical analyses to the Supporting information section and remove Figure 5 from the manuscript (L. 329-332).

*Comment#4:*

*With multiple conditions (group, task, phase) it is sometimes difficult to follow the results. The table refers to ‘task’ but the text refers to ‘condition’ when referring to ‘Prediction’ or ‘Control’. I would suggest to keep using the same term to refer to either the task or the condition.*

*In line with this, it is also not easy to compare the actual results to each other (e.g. fixation duration of experts an novices in the prediction task during the rally phase). Although this information is in the text, I think an extra table (perhaps as supplementary info) would benefit the clarity of the results. I would suggest to move table 3 to appendix and replace it by a similar table with the average +- SD results.*

A more precise phrasing concerning the issue of task and condition was implemented. We made sure to only refer to “Prediction” and “Control” as conditions (of the task) in the revised manuscript. In naming the corresponding LMM factor “Task” we kept continuity with the naming scheme used in our previous study involving the same task [6]. We adapted the corresponding part in the Statistical analysis section to clarify that the factor “Task” refers to the two task conditions (L. 335-337). We also adopted the suggestion to replace Table 3 (L. 466-468) with a table providing descriptive results and moving the original Table 3 (now Table A) to the Supplementary Material (L. 433-435).

*Comment #5*

*The experts in the prediction condition during rally had a Fixation duration of 1041 ms. However, their Fixation rate in this phase and condition was 1.21 fix/sec. How is an average frequency of more than 1 fixation per second possible if the average fixation duration is more than 1 second long?*

This is a crucial methodological aspect that is necessary to clarify, we thank you for making us aware of it. This discrepancy between fixation rate and fixation duration can be explained by the fact that the duration of a fixation was defined as the timeframe from the onset of the fixation until the time point when a new saccade or blink occurred. This means that the last fixation of each phase stretched into the subsequent phase. Therefore, fixation rate and fixation duration have to be interpreted with this circumstance in mind (i.e., fixation rate = rate of unique fixation onsets during the specific time frame of the phase; fixation duration = average duration of fixations with onset during the phase, irrespective of phase length). We see the caveat of this procedure, namely that fixation durations cannot be solely attributed to one phase. However, this procedure has the advantage that by attributing fixations to the phase of their onset the temporal alignment with the cognitive trigger causing the fixation is maintained. We deemed this procedure was better suited than applying a fixation-splitting approach (thereby artificially inflating the number of fixations) or excluding cross-phase fixations entirely (thereby causing considerable loss of data due to the rather narrow time frames in our experiment). In the Preprocessing section we have already discussed the issue of fixations with durations stretching across the end of a whole trial, i.e., unusually long fixations. Such fixations as well as ones with estimated gaze points outside the screen dimensions were excluded from the analysis, which only accounted for a very small percentage of our analyzed fixation data (0.23%; L. 314-316). In the revised manuscript we now extended the Preprocessing section, explaining our approach regarding fixation durations during phase transitions (L. 287-291).

*Comment #6:*

*The authors already added the results of accuracy (from another paper: Kanatschnig et al. [2]) to the discussion, but I believe that it would be better to report this as results as well. However, I agree with the authors that this should not be elaborated on as it its discussed in another paper.*

We also made the consideration of adding more information and data regarding the behavioral task results of the experiment to the present manuscript. However, as you will understand, this would produce the issue of double publication, which is the main reason why we chose not to go more in depth into the behavioral data in this manuscript. However, we now included a reference to the open data repository [7] where the data as well as analysis code from our previous publication [6] can be inspected (L. 45-47).

*Comment #7:*

*L540: “It is necessary to emphasize that we were only able to observe these dynamic 541 patterns results through the distinction between Preparation and Rally phases, but also through 542 the inclusion of amateurs as an intermediate level group in our experimental design.”*

*Missing the word ‘not’: … were not only able to observe … ?*

Thank you very much for this hint, this was corrected (L. 574-576).

*Comment #8:*

*The authors should also refer to a recent study with a very similar experimental design:*

*Zhu, R., Zou, D., Wang, K., & Cao, C. (2024). Expert performance in action anticipation: visual search behavior in volleyball spiking defense from different viewing perspectives. Behavioral Sciences, 14(3), 163.*

Thank you for this recommendation. We evaluated and incorporated this reference into our manuscript as we deem it to fit the topic of our investigation well (L. 89-91).

# Journal Requirements

*If the reviewer comments include a recommendation to cite specific previously published works, please review and evaluate these publications to determine whether they are relevant and should be cited. There is no requirement to cite these works unless the editor has indicated otherwise.*

Literature which was recommended by reviewers was evaluated on their appropriateness for citation.

# Additional Changes

- Figure 1 and its caption have been reworked to improve the structure of the figure and its supporting text.
- The file “S6_Tables” including supplementary tables was added as Supporting information upon Reviewer 3’s recommendation.
- The file “S5_Code” was expanded to include additional statistical analyses upon Reviewer 2’s recommendation.
- Figure 5 was removed from the manuscript upon Reviewer 3’s recommendation.
- The Acknowledgements section has been rephrased to revoke the notion that the involvement of the doctoral consortium COLIBRI was of financial nature.

# References

1.	Faubert J. Professional athletes have extraordinary skills for rapidly learning complex and neutral dynamic visual scenes. Sci Rep. 2013;3: 1154. doi:10.1038/srep01154

2.	Dunn MJ, Alexander RG, Amiebenomo OM, Arblaster G, Atan D, Erichsen JT, et al. Minimal reporting guideline for research involving eye tracking (2023 edition). Behav Res Methods. 2023;56: 4351–4357. doi:10.3758/s13428-023-02187-1

3.	Walcher S, Korda Ž, Körner C, Benedek M. The effects of type and workload of internal tasks on voluntary saccades in a target-distractor saccade task. Wang J, editor. PLoS One. 2023;18: e0290322. doi:10.1371/journal.pone.0290322

4.	Foulsham T, Kingstone A. Fixation-dependent memory for natural scenes: An experimental test of scanpath theory. J Exp Psychol Gen. 2013;142: 41–56. doi:10.1037/a0028227

5.	Ladyka-Wojcik N, Liu ZX, Ryan JD. Unrestricted eye movements strengthen effective connectivity from hippocampal to oculomotor regions during scene construction. Neuroimage. 2022;260: 119497. doi:10.1016/j.neuroimage.2022.119497

6.	Kanatschnig T, Schrapf N, Leitner L, Wood G, Körner C, Tilp M, et al. EEG theta and alpha oscillations during tactical decision-making: An examination of the neural efficiency hypothesis in volleyball. PLoS One. 2025;20: e0318234. doi:10.1371/journal.pone.0318234

7.	Kanatschnig T, Kober SE, Leitner L, Tilp M, Schrapf N, Körner C, et al. Neural efficiency during tactical decision-making in volleyball. Open Science Framework; 2022. doi:10.17605/OSF.IO/CK5ZU
